# Saccharated Calomel, Again

**Published:** 1874-04

**Authors:** M. R. May

**Affiliations:** Athens, Tenn.


Those of our readers—and we trust there are very many such—who have been led to the adoption into their daily practice of the elegant little powder of *saccharated calomel*, to which we called attention last year in this journal, we feel that no apology is needed for presenting the subjoined letter from one of our correspondents touching its history. However ancient may have been the combination of sugar with calomel we are of opinion that its very remarkable cholagogue power, in minute doses, is of modern discovery. The fact that one-half grain of calomel in intimate combination with one grain of loaf sugar is capable of inducing three to five free, billious evacuations from the bowels of a strong man, has been known to us but a few brief years, and probably not longer to the great mass of our readers. We have not doubted that the combination of calomel with sugar is old; yes, very old; nor have we any doubt that the modification of therapeutical effect by trituration of this as well as dover's powder and various other powders is an idea as old as Hahnemann, but the fact that a minute dose possesses so great cholagogue power as to induce the active billious purgation we have mentioned, we have not as yet been able to trace to the knowledge of any one behind the experience of Dr. John M. Johnson, who assures us that he has been observing it in his practice for thirty years; a period reaching back beyond the publication of Dr. Horace Green, of New York :

*Editor Atlanta Medical and Surgical Journal:*

In your August number you published a paper on saccharated calomel in which we find the following: "It is proper that I should disclaim the discovery of this combination, for which I am indebted to my friend Dr. Miller, of Atlanta, Ga. Whether it is original with him or not, I am unable to say." In the September and October numbers, in which you say that, "we are happy to be able now to state, upon the authority of the originator himself, that this honor is justly due to Dr. John M. Johnson, of Atlanta, Ga." A correspondent in the January number, 1874, calls your attention to a book entitled "Selections from Private Prescriptions of Living American Practitioners, by Horace Green, M.D., LL.D., New York, 1858," page 77, in which saccharated calomel was used. Please let me premise that whilst I have no design to impugn the motives of any gentleman, nor to attack his integrity, but (as you say, to "accord honor to whom honor is due") to show that by consulting Hamilton on Purgatives, 5th Edinburg edition, Appendix viii, page 76, you will perceive that in a case of

"Chorea Sancti Vite," he prescribed saccharated calomel nearly seventy years ago. It is true that his doses were larger, though he enjoined intimate admixture by thorough trituration (tere intime) a very important matter in your estimation, to render the combination efficacious. I will report the case and you can draw your own conclusions.

ROYAL INFIRMARY, December 5, 1804.

David Anderson, æt. 8, is subject to violent, irregular and involuntary motions of the muscles of the head, eyes, lower jaw, abdomen, both superior and inferior extremities, etc. "*Habeat bolum e jalapa cum mercurio.*" Dec. 6. Refused the bolus:

℞.—Submuriatis Hydrargyri Scrupulum,
Sacchari Albi Drachmam.

Tere intime, et divide in doses duodecim, quarum sumat unam, omni bihorio."

M. R. MAY, M.D.

ATHENS, TENN., March, 1874.

---

WE learn that Dr. James C. Harris, of Wetumpka, Ala., has been elected an *honorary member* of the Medical and Surgical Society of Montgomery, in recognition of his meritorious contributions to medical science.

